# Full-Length Genome Sequence of Porcine Deltacoronavirus Strain USA/IA/2014/8734

**DOI:** 10.1128/genomeA.00278-14

**Published:** 2014-04-10

**Authors:** Ganwu Li, Qi Chen, Karen M. Harmon, Kyoung-Jin Yoon, Kent J. Schwartz, Marlin J. Hoogland, Phillip C. Gauger, Rodger G. Main, Jianqiang Zhang

**Affiliations:** a Department of Veterinary Diagnostic and Production Animal Medicine, College of Veterinary Medicine, Iowa State University, Ames, Iowa, USA; b Murphy-Brown LLC, Algona, Iowa, USA

## Abstract

Porcine deltacoronavirus (PDCoV) was detected in feces from diarrheic sows during an epidemic of acute and transmissible diarrhea. No transmissible gastroenteritis virus or porcine epidemic diarrhea virus was detected. The PDCoV USA/IA/2014/8734 from the herd was sequenced for full-length genomic RNA to further characterize PDCoV in U.S. swine.

## GENOME ANNOUNCEMENT

Coronaviruses (CoVs) are enveloped, single-stranded, positive-sense RNA viruses belonging to the order *Nidovirales* and the family *Coronaviridae*. Three genera, *Alphacoronavirus*, *Betacoronavirus*, and *Gammacoronavirus*, were proposed to replace the traditional group 1, 2, and 3 coronaviruses (1–3). The fourth genus, *Deltacoronavirus*, has recently been described by a research group in Hong Kong (4, 5). By use of a molecular tool, they detected deltacoronaviruses in various species, including at least nine avian CoVs and two porcine CoVs (HKU-15-44 and HKU-15-155).

In late February of 2014, we received a submission for diagnostic investigation from a 2,500-sow herd in Iowa with a history of acute severe diarrhea. The epidemic started in one breeding barn and progressed throughout the breeding and gestation barns over a 7-day period. Acute diarrhea started in the farrowing rooms (sows and piglets) approximately 8 to 9 days after the initial onset of clinical signs in the breeding barn. Viral enteritis was suspected based on clinical impression and gross and microscopic lesions. Molecular testing did not detect porcine epidemic diarrhea virus, transmissible gastroenteritis virus, or porcine rotaviruses. Bacterial culture yielded mixed and inconsistent populations of expected flora with no significant pathogens consistently identified. Unexpectedly, all fecal samples tested positive for porcine deltacoronavirus (PDCoV) by a pan-*Coronaviridae* PCR (6) followed by sequencing confirmation of PCR amplicons, as well as by a PDCoV-specific real-time reverse transcription (RT)-PCR assay with cycle threshold (C_*T*_) values ranging from 14 to 19.

Since the full-length genomic sequences of PDCoV in U.S. swine have not been previously reported, complete genomic sequencing of PDCoV (USA/IA/2014/8734) was attempted using next-generation sequencing technology on an Illumina MiSeq platform following the procedures established in our laboratory (7). Sequences were mapped to all known coronaviruses and *de novo* assembled and then analyzed using the DNAStar Lasergene 11 Core Suite.

The genomic sequence of the PDCoV USA/IA/2014/8734 strain is 25,422 nucleotides (nt) in length, excluding the 3′ poly(A) tail. The genomic organization of this U.S. PDCoV is similar to what was previously described for PDCoV HKU15-44 and HKU15-155 (4). The genome arrangements are as follows: 5′ untranslated region (UTR), open reading frame 1a/1b (ORF1a/1b), spike (S), envelope (E), membrane (M), nonstructural protein 6 (NS 6), nucleocapsid (N), nonstructural protein 7 (NS 7), and 3′ UTR.

The genome of PDCoV USA/IA/2014/8734 has 271 nt differences (98.9% nt identity) from the HKU15-44 strain (GenBank accession no. JQ065042) and 209 nt differences (99.2% nt identity) from the HKU15-155 strain (JQ065043). The majority of nt differences were located in the ORF1a/1b and S genes. There were no insertions or deletions between USA/IA/2014/8734 and HKU15-44. However, compared to HKU15-155, USA/IA/2014/8734 has six nucleotide insertions (a 3-nt AAT insertion at positions 19477 to 19479, corresponding to the S gene, and a 3-nt GTT insertion at positions 25047 to 25049, corresponding to the 3′ UTR).

The PDCoV USA/IA/2014/8734 sequence data will facilitate future research on the epidemiology and evolutionary biology of PDCoVs in U.S. swine. Further study remains to be conducted to determine the clinical significance of PDCoV.

### Nucleotide sequence accession number.

The complete genome sequence of PDCoV strain USA/IA/2014/8734 has been deposited in GenBank under the accession number KJ567050.
